# Morphological and genomic data from Belgian naturalised populations reinstate the enigmatic *Lathyrus platyphyllos* (Fabaceae) as a distinct European species

**DOI:** 10.3897/phytokeys.273.180869

**Published:** 2026-04-23

**Authors:** Filip Verloove, Sander de Backer, Yves Bawin, Quentin Groom, Frederik Leliaert, Sofie Meeus, Steven B. Janssens

**Affiliations:** 1 Meise Botanic Garden, Nieuwelaan 38, Meise, Belgium Meise Botanic Garden Meise Belgium; 2 Leuven Plant Institute, Department of biology, KU Leuven, Kasteelpark Arenberg 31, Leuven, Belgium Hasselt University Hasselt Belgium; 3 Hasselt University, Faculty of Science, Agoralaan building D, 3590 Hasselt, Belgium KU Leuven Leuven Belgium

**Keywords:** Belgium, *Lathyrus sylvestris–latifolius* complex, non-native flora, SNP phylogenomics, species delimitation

## Abstract

The genus *Lathyrus* L. (Fabaceae) is represented in Belgium by several native and alien species, amongst which the *Lathyrus sylvestris*–latifolius complex has long posed taxonomic difficulties. Since the 1940s, plants with intermediate morphological characters between *L. sylvestris* and *L. latifolius* have been observed in Belgium, combining the smaller corollas of the former with the broader, rounded leaflets of the latter. To clarify their identity, we conducted phylogenetic analyses of four chloroplast markers (*trnS-G*, *trnL-F*, *rbcL*, *trnH-psbA*) and the nuclear rDNA internal transcribed spacer (ITS), complemented by genome-wide SNP data obtained through DArTseq genotyping for 20 individuals representing *L. latifolius*, *L. heterophyllus*, *L. sylvestris* and the enigmatic Belgian specimens.

Phylogenetic analyses, based on the five markers, together with analysis of the SNP data (Principal Coordinates Analysis, Maximum Likelihood phylogenetic reconstruction and multispecies coalescent species delimitation), all revealed four well-supported genetic lineages corresponding to these taxa. The enigmatic specimens form a monophyletic clade sister to *L. sylvestris*, genetically distinct from both *L. heterophyllus* (including var. *unijugus*) and *L. latifolius*. These results exclude hybrid origin and support recognition of these plants as an independent evolutionary lineage, which can, thus, be regarded as a distinct species.

In light of both morphological and molecular evidence, we reinstate the name *Lathyrus platyphyllos* (Retz.) W.D.J.Koch for this taxon. *Lathyrus platyphyllos* differs from *L. sylvestris* by its broader leaflets with rounded apices and its wider stipules and stem wings. The species is non-native in Belgium, first recorded in 1943 and now locally naturalised along railway lines and other disturbed sites. Recognition of *L. platyphyllos* restores taxonomic clarity within the *L. sylvestris*–latifolius complex and highlights the need for further study of its native range, introduction pathways and potential invasiveness.

## Introduction

The genus *Lathyrus* L. (Fabaceae) comprises approximately 180 species distributed mainly across temperate and subtropical regions, with a few extending into tropical montane zones (Kenicer et al. 2005). In Belgium, eight species are considered native or archaeophytic: *L. aphaca* L., *L. hirsutus* L., *L. linifolius* (Reichard) Bässler, *L. nissolia* L., *L. pratensis* L., *L. sylvestris* L., *L. tuberosus* L. and possibly *L. japonicus* Willd. (Verloove and Van Rossum 2024). However, the native status of several of these remains uncertain, as many historical records may represent naturalised introductions (Lawalrée 1963). In addition, twelve unquestionably alien species have been reported from Belgium, of which only *L. latifolius* is now widely naturalised (Verloove 2006). Despite this diversity, the genus has received little modern taxonomic study in the region.

Amongst Belgian representatives, the *Lathyrus sylvestris*–latifolius complex is particularly problematic. These perennial species share similar vegetative morphology, with Belgian populations consistently showing winged stems, one pair of leaflets per leaf and multi-flowered inflorescences and have long been regarded as taxonomically challenging (Valero and Hossaert-McKey 1991; Kenicer and Norton 2008). The complex is largely European in distribution, with *L. sylvestris* extending further into parts of western Asia, while *L. heterophyllus* and *L. latifolius* have more restricted ranges within Europe. Historically, authors differed in their approach to the morphological variation within the *Lathyrus sylvestris* complex. For instance, Ginzberger (1896) applied a very narrow species concept, recognising numerous taxa that are now largely treated as synonyms of *L. sylvestris* or *L. latifolius*. By contrast, Fiori (1925) followed a broader species concept, treating forms corresponding to what are now recognised as *L. platyphyllos*, *L. latifolius* and *L. heterophyllus* as varieties of a single, variable species, *L. sylvestris*.

Since the 1940s, plants exhibiting intermediate morphology between *L. sylvestris* and *L. latifolius* have increasingly been observed in Belgium. These plants possess the smaller corollas and flower colouration typical of *L. sylvestris* (≤ 20 mm), but combine them with vegetative traits more characteristic of *L. latifolius*, notably broad, rounded leaflets up to 40 mm wide and a vigorous climbing habit. Their systematic position has long remained ambiguous (Verloove and Van Rossum 2024).

Two main hypotheses have been proposed for their identity. Some authors have suggested that these plants correspond to *Lathyrus heterophyllus* L., a polymorphic European species that includes unijugate (having one pair of leaflets) variants described as var. *unijugus* W.D.J. Koch (Melderis 1957; Stace 2019). Alternatively, they have been referred to *L. sylvestris* var. platyphyllos Retz. (syn. subsp. platyphyllos (Retz.) Hartm.), a broad-leaved taxon of uncertain rank and distribution. Koch (1843) recognised the close resemblance between both entities, noting that “*L. heterophyllus* var. *unijugus* appears identical to *L. platyphyllos* Retz.”. Most modern databases, including Euro+Med Plantbase and POWO (2024), now treat these taxa as synonyms – respectively of *L. heterophyllus* and *L. sylvestris* – leaving their actual identity unresolved.

In Belgium, these enigmatic plants have been known since at least 1943 and are now naturalised at several sites, particularly along railway lines and disturbed habitats. Their combination of distinctive morphology, recent establishment and expansive growth behaviour raises questions about their taxonomic identity and possible ecological impact.

To clarify their systematic position, we performed a genomic study to address the following questions. First, are the morphologically intermediate Belgian plants genetically distinct from *L. sylvestris*, *L. heterophyllus* and *L. latifolius*? If so, to which of these species are they most closely related? Second, are *L. sylvestris* var. platyphyllos and *L. heterophyllus* var. *unijugus* genetically distinct or do they represent a single taxon? Lastly, what taxonomic rank, if any, should be assigned to these plants?

## Materials and methods

### Data collection and processing

Sampling

A total of 20 individuals (mostly from Belgium with five specimens from Sweden, Italy and Switzerland) were included in the study, comprising five individuals each of *L. latifolius*, *L. heterophyllus*, *L. sylvestris* and the Belgian *Lathyrus* specimens (hereafter called the “*Lathyrus* indet.” specimens). Vouchers were deposited in the Herbarium of Meise Botanic Garden (BR) and are available online via https://www.botanicalcollections.be/ and the Global Biodiversity Information Facility (GBIF) (Meise Botanic Garden 2024; GBIF.org 2025). Collection details are provided in Suppl. material 1: table S1.

DNA extraction and sequencing

Genomic DNA was extracted from dried leaf tissue using a modified cetyltrimethylammonium bromide (CTAB) protocol (Doyle and Doyle 1987). DNA concentration and purity were assessed with a Fragment Analyzer (Agilent Technologies, Inc.).

For the Sanger sequencing approach, amplification reactions of the plastid markers *trnL-F* (Taberlet et al. 1996), *trnS-G* and *trnH-psbA* (Hamilton 1999) and *rbcL* (Savolainen et al. 2000), as well as the nuclear ribosomal ITS region (White et al. 1990), were performed on a GeneAmp PCR System 9700 (Applied Biosystems). Following an ExoSAP purification protocol, PCR products were sent for sequencing to Macrogen Europe (Amsterdam, the Netherlands). Newly-generated sequences were deposited in the European Nucleotide Archive (ENA) under project accession PRJEB103024.

Diversity Arrays Technology sequencing or DArT sequencing (Canberra, Australia) was performed with the DArTseq platform (Kilian et al. 2012), which combines enzyme-based complexity reduction (PstI and MseI) with Illumina NovaSeq 6000 sequencing. All raw sequencing data together with the Sanger sequences have been deposited in the European Nucleotide Archive (ENA) in the project PRJEB103024.

Sequence processing

Assembly of Sanger sequencing data was conducted in Geneious Prime 2025.1.2 (Biomatters, New Zealand). Automatic alignments were produced with MAFFT (Katoh et al. 2002) using the E-INS-i algorithm, a 100PAM/k=2 scoring matrix, a gap-opening penalty of 1.3 and an offset value of 0.123. Alignments were subsequently inspected and manually refined in Geneious Prime. Potential conflicts between data partitions were assessed visually, focusing on incongruent relationships supported by ≥ 70% bootstrap values (hard vs. soft incongruence) (Johnson and Soltis 1998).

Raw reads retrieved from DArT were quality-checked using FastQC and trimmed to remove barcodes and restriction enzyme cut-site remnants with Cutadapt v.3.5 (Martin 2011) as part of GBprocesS v.4.0.0 (Schaumont 2024). Variation in barcode length was addressed by 3’-trimming reads with a barcode shorter than the maximum barcode length with Cutadapt. Reads shorter than 20 bp after trimming were removed. Additionally, all reads with more than five ambiguous nucleotide calls (Ns), an average quality score below 25 or internally intact PstI or MseI restriction sites were discarded with GBprocesS. Reads were mapped against the *Lathyrus tuberosus* L. reference genome (Flood et al. 2022) using BWA-MEM v.0.7.17-r1188 (Li and Durbin 2009) with default settings. All reads were tagged with read groups using the AddOrReplaceReadGroups module from Picard v.3.3.0 (Broad Institute 2019). Single nucleotide polymorphisms (SNPs) were called in mapped reads using the UnifiedGenotyper module in the Genome Analysis Toolkit (GATK) v.3.7 (McKenna et al. 2010). SNP calls were subsequently filtered for a minimum quality score of 20, a minimum mean read depth of 5 and a minimum minor allele frequency of 5% with a custom python script (de Backer 2025). Only polymorphic and biallelic SNPs were retained as part of the same custom python script.

### Data analysis

#### Similarity analysis

To obtain a genetic distance matrix of Jaccard similarity coefficients, variation in SNPs and restriction site polymorphisms at 5’ and 3’ locus margins (SMAPs) was converted into haplotype calls using the SMAP software package (Schaumont et al. 2022). SMAP variation was determined using SMAP *delineate* with a minimum stack depth of 5 and minimum cluster depth of 10, whereas loci present in less than 5% of the samples were ignored. SMAP haplotype-sites were used to call read-backed haplotypes. The following settings were used for haplotype calling: loci with a minimum of 20 reads and a minimum MAPQ score of 20 and haplotypes with a minimum frequency of 5% in a locus. Haplotypes containing insertions or deletions were removed. Next, haplotype frequencies were converted into discrete dominant calls (0 or 1) with a frequency bound interval of 20%. The Jaccard similarity coefficient (J) (Jaccard 1912) and inversed distance (1 – J) were calculated using SMAP *relatedness pairwise* (Bawin et al. 2025). The resulting genetic distance matrix was used to perform a Principal Coordinates Analysis (PCoA) with the *wcmdscale* function from the VEGAN package v.2.6-4 (Oksanen et al. 2023) in R v.4.3.1 (R Core Team 2024).

Phylogenetic analysis

Maximum Likelihood (ML) analyses with Sanger sequences were conducted with IQ-TREE v.2.4.0 (Nguyen et al. 2015; Minh et al. 2020) using the best-fit substitution model (F81+F) selected according to the Bayesian Information Criterion (BIC) (Kalyaanamoorthy et al. 2017). Node support was assessed with 1,000 ultrafast bootstrap replicates (Hoang et al. 2018).

For each polymorphic locus in the DArTseq data, a nucleotide alignment of consensus sequences was constructed with the SMAPapp-Alignment.py script available from the SMAPapps GitLab project (https://gitlab.com/ybawin/smapapps). The consensus sequence of each locus consisted of both SNPs and invariable sites. Consensus sequences with only invariable sites and ambiguous SNP calls were discarded. A Maximum Likelihood phylogenetic tree was constructed for each locus separately with IQ-Tree2 (Minh et al. 2020) using the ModelFinder tool (Kalyaanamoorthy et al. 2017) to select the best fitting substitution model for each locus, based on the corrected Akaike Information Criterion (AICc). Branch support values were calculated, based on 1000 non-parametric bootstrap replicates. Finally, all locus consensus trees were used to reconstruct a global consensus tree with ASTRAL-III (Zhang et al. 2018). Trees were visualised with FigTree v.1.4.4 (Rambaut 2018) and *L. latifolius* was chosen as the outgroup, based on a phylogenetic study by Schaefer et al. (2012).

Molecular species delimitation

We conducted a species delimitation analysis in a Bayesian framework with the biallelic SNP data using SNAPP v.1.6.1 (Bryant et al. 2012) as implemented in BEAST v.2.7.7 (Bouckaert et al. 2014). By implementing a full coalescent model, SNAPP infers species phylogenies from unlinked biallelic data, such as a large SNP dataset (Bryant et al. 2012). Analysis parameters were set in BEAUTi v.2.7.7 using the SNAPP template. The MCMC chain length was set to 10 million, sampling every 5000 generations, for two independent analyses. The two runs were combined with LogCombiner v.2.7.3 (Bouckaert et al. 2014), with a burn-in of 10% and used as input for the SpeciesDelimitationAnalyser tool (Jones et al. 2015; Jones 2017) to process the distribution of species assignments.

## Results

### DArTseq sequencing metrics and variant retention

The high density service on the *Lathyrus* sequencing platform at DArT yielded an average of 3.6 million reads per sample. The read data in each sample covered on average 0.08% of the *L. tuberosus* L. reference genome (Flood et al. 2022), with an average mapping percentage of 60.69%.

Initial variant calling identified a total of 694,752 SNPs. After applying quality filters, 78,200 high-quality SNPs were retained in 25,359 loci generally evenly distributed across the reference genome. There was an average of 72.69% missing data per sample.

### Similarity analysis

**Figure 1.**
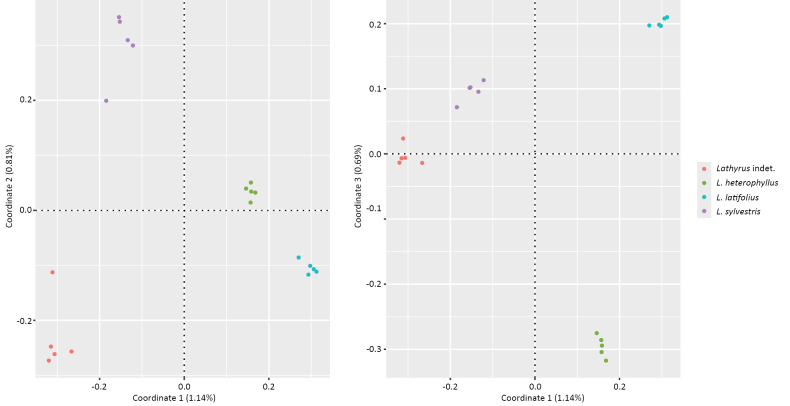
Principal Coordinates Analysis (PCoA) of *Lathyrus* specimens, based on SMAP-derived haplotypes. (left) The first and second coordinates show clear separation of all four major genetic clusters. (right) The first and third coordinates place the *Lathyrus* indet. specimens closest to *L. sylvestris*, suggesting a stronger genetic relationship.

The Jaccard genetic distance matrix indicated that the *Lathyrus* indet. specimens are genetically distinct from *L. heterophyllus* and showed the highest pairwise similarity with *L. sylvestris* (Suppl. material 1: fig. S2). This pattern was further corroborated by Principal Coordinates Analysis (PCoA) (Fig. 1), which provided a model-free ordination of genetic distances in multivariate space. In the PCoA plot, the *Lathyrus* indet. specimens consistently clustered closest to *L. sylvestris* along the first and third principal coordinates, reflecting their relative genetic proximity. Each of the four sampled groups (*L. latifolius*, *L. heterophyllus*, *L. sylvestris* and the *Lathyrus* indet. specimens) formed well-separated, non-overlapping clusters across the three main coordinate axes. This indicates strong genetic structuring amongst taxa and supports the presence of four genetically distinct lineages. Notably, the *L. heterophyllus* samples included two collections from Sweden (BR0000029726080 and BR0000029726066) exhibiting unijugate leaves (var. *unijugus*). These Swedish samples clustered tightly with the rest of *L. heterophyllus* (var. heterophyllus) and no genetic differences were detected. While this suggests that the unijugate form is not strongly differentiated in our dataset, we cannot exclude a genetic basis for leaflet number. Importantly, this also confirms that the cryptic Belgian specimens cannot be assigned to var. *unijugus*, further supporting their status as a separate genetic lineage. Although the first three principal coordinates explained only 2.64% of the total genetic variance, the clear separation amongst groups demonstrates that even limited variance can effectively resolve taxonomic boundaries in this dataset.

### Phylogenetic analyses

The concatenated alignment of the Sanger sequencing dataset consisted of four chloroplast markers and one nuclear ribosomal ITS region. The nuclear ITS alignment contained 573 characters, of which four were variable. For the plastid markers, *psbA-trnH* comprised 248 characters (10 variable), *rbcL* 702 characters (2 variable), *trnL-F* 602 characters (4 variable) and *trnS-G* 756 characters (8 variable). No significant incongruence was detected through visual comparison of the single-marker topologies (Suppl. material 1: fig. S1). The five loci were, therefore, concatenated for all subsequent analyses.

**Figure 2.**
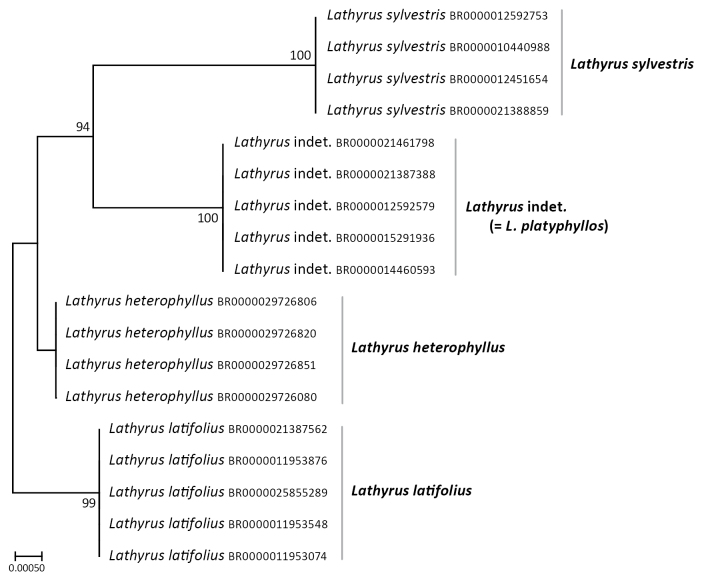
Maximum-Likelihood (IQ-Tree) tree inferred from the concatenated alignment (ITS, *trnS*-*G*, *trnL*-*F*, *rbcL*, *trnH*-*psbA*), showing a well-supported *Lathyrus* indet. clade. ML ultrafast bootstrap values (≥ 90%) are indicated at each branch. Meise Botanic Garden herbarium barcodes are indicated next to the taxon names.

Maximum Likelihood analysis of the combined dataset recovered four clades corresponding to *Lathyrus sylvestris*, *Lathyrus* indet., *Lathyrus latifolius* and *Lathyrus heterophyllus*. Of these, *Lathyrus heterophyllus* was unsupported, whereas the other three were highly supported (ML ultrafast bootstrap values: 100, 100 and 99, respectively). Despite the relatively low number of variable plastid characters and variation in the completeness of plastid sampling, this had no impact on the resulting topology, as the nuclear ITS dataset—although containing few variable sites— provides consistent signal supporting the same relationships (Fig. 2).

**Figure 3.**
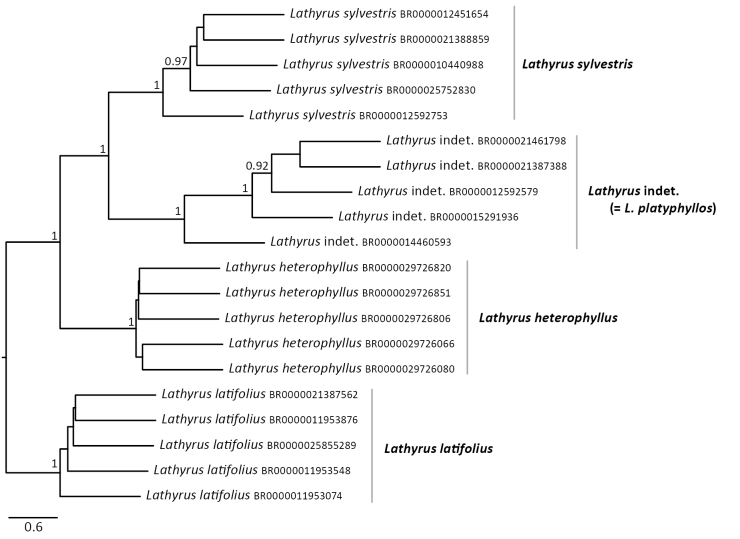
Consensus species tree as inferred by ASTRAL-III using 10,983 Maximum Likelihood locus trees generated with IQ-Tree. Node values indicate local posterior probabilities. The tree shows a well-supported monophyletic group for the *Lathyrus* indet. specimens.

A clear genetic distinctiveness of the *Lathyrus* indet. specimens was found in the consensus phylogenetic tree, based on the DArTseq loci. A total of 10,983 Maximum Likelihood locus trees were used to infer the species tree. The resulting species tree (Fig. 3) shows that these specimens form a monophyletic clade sister to *L. sylvestris*, indicating that they share a more recent common ancestor with *L. sylvestris* than with *L. heterophyllus* or *L. latifolius*. The clade comprising the *Lathyrus* indet. and *L. sylvestris* is, in turn, sister to *L. heterophyllus*. The inferred topology is fully congruent with the separation observed in the Jaccard similarity matrix (Suppl. material 1: fig. S2) and PCoA (Fig. 1). The backbone of the topology is well-supported, reflecting a strong, clear signal from deep divergences across genome-wide loci. In contrast, shallow divergences (e.g. within species) are too weak, leading to conflicting signals and incomplete lineage sorting that ASTRAL cannot confidently resolve.

### Molecular species delimitation

Species delimitation analysis using SNAPP (Bryant et al. 2012) in BEAST2 (Bouckaert et al. 2014), based on biallelic SNP markers under the multispecies coalescent model, identified four distinct species-level lineages (Suppl. material 1: fig. S3). This delimitation scenario was recovered by the SpeciesDelimitationAnalyser (Jones et al. 2015; Jones 2017) in 99.9% of the posterior distribution (Suppl. material 1: table S2), providing very strong support for the existence of four evolutionary independent lineages within the sample set. Only a small proportion of trees (0.1%) supported an alternative scenario in which *L. sylvestris* and *L. heterophyllus* were not resolved as separate species.

## Discussion

The results of this study provide strong evidence for the hypothesis that the enigmatic *Lathyrus* specimens from Belgium represent a genetically distinct lineage, separate from the closely-related species *L. sylvestris*, *L. latifolius* and *L. heterophyllus*. Multiple lines of evidence— including phylogenetic inference, model-free ordination and pairwise genetic distance calculation— consistently place the *Lathyrus* indet. specimens within a well-supported and cohesive genetic cluster, with no signs of recent admixture. This pattern suggests that these populations are not the product of recent hybridisation events, but, instead, constitute an independent evolutionary lineage that warrants recognition as a distinct species.

Phylogenetic analyses further clarify the evolutionary relationships of the *Lathyrus* indet. lineage. This topology was recovered in both the Maximum Likelihood framework and the coalescent-based SNAPP species tree, with strong statistical support. The extremely low proportion of SNAPP trees (0.1%) supporting a collapse of *L. sylvestris* and *L. heterophyllus* into a single species underscores their genetic distinctiveness.

Notably, the *L. heterophyllus* samples included two collections from Sweden exhibiting unijugate leaves (var. *unijugus*). In our analysis, these samples clustered tightly with the rest of *L. heterophyllus* (var. heterophyllus) and no genetic differences were detected. This suggests that the unijugate form is not strongly differentiated, though it may still represent a valid infraspecific taxon. This observation further confirms that the cryptic *Lathyrus* indet. specimens do not belong to var. *unijugus*, reinforcing their status as a separate genetic lineage.

These molecular findings align with previously observed morphological ambiguities within this species complex. Intermediate or overlapping phenotypes, particularly between *L. sylvestris* and *L. latifolius*, have long challenged taxonomic resolution. The use of coalescent-based species delimitation (SNAPP), which is particularly effective at detecting recent divergence using biallelic SNP data (Bryant et al. 2012), proved critical in this context. Although more conventional DNA barcoding markers were also able to separate these species, SNAPP provided a finer-scale resolution of subtle genetic discontinuities between closely-related taxa, demonstrating its utility in addressing cryptic diversity in morphologically conserved groups.

Taken together, our results strongly support the recognition of the *Lathyrus* indet. specimens as a distinct, non-native *Lathyrus* taxon in Belgium. The establishment and spread of *L. platyphyllos* in Belgium may have significant ecological implications, including competition with native *Lathyrus* species and changes to habitat structure due to its vigorous growth. Further research is needed to clarify its native range, pathways of introduction and potential for spread— especially given its apparent naturalisation and resemblance to known invasive species such as *L. latifolius*.

## Taxonomic treatment

### Reinstatement of *Lathyrus platyphyllos*

#### Lathyrus platyphyllos

Taxon classificationPlantaeFabalesFabaceae

(Retz.) W.D.J.Koch, Syn. Fl. Germ. Helv. ed. 2: 443 (1843)

9341782B-6A11-558C-A19B-F8EBCDD5230D

urn:lsid:ipni.org:names: 77371641-1

≡ Lathyrus sylvestris var. platyphyllos Retz., Fl. Scand. Prodr., ed. 2.: 170 (1795).
≡ Lathyrus sylvestris subsp. platyphyllos (Retz.) Hartm., Sv. Norsk Excurs.-Fl. 102 (1846).
≡ Lathyrus sylvestris f. platyphyllos (Retz.) Nordh., Norsk Fl. [Nordhagen]: 381 (1940).

##### Holotype.

t. DCCLXXXV in *Flora Danica* s.d. (1787?). The protologue of *Lathyrus platyphyllos* refers exclusively to this illustration, with no mention of any specimens. Following ICN Art. 9.1–9.2 and in line with the guidelines discussed by McNeill (2014), this plate is treated here as the holotype. While it is theoretically possible that Retzius consulted additional, uncited elements, this illustration represents the only verifiable original element.

##### Diagnosis.

**Figure 4.**
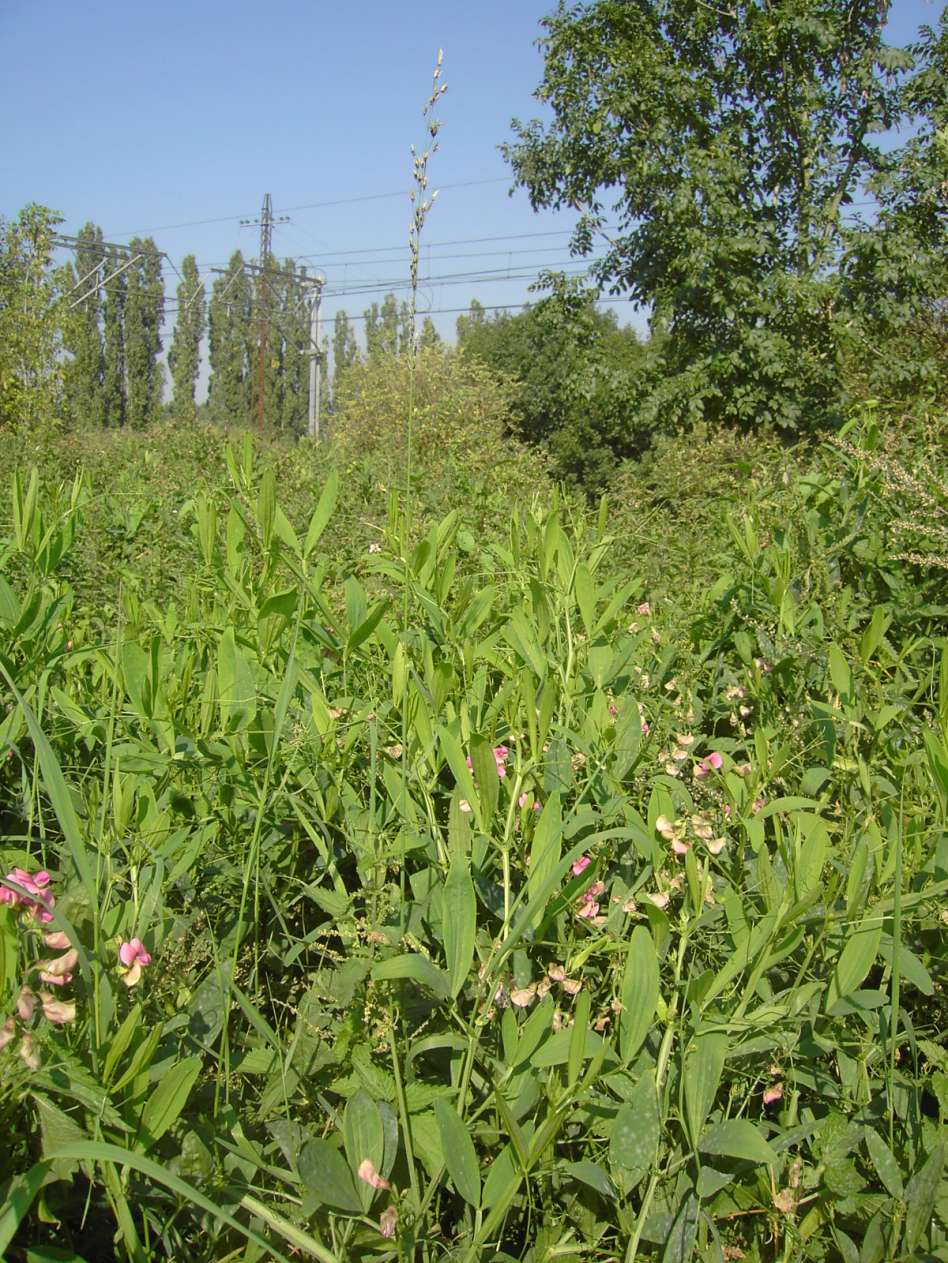
*
Lathyrus platyphyllos* in Kortemark, Belgium, 3 September 2011, F. Verloove. In general habit (vigour, leaflet width), this species strongly resembles *L. latifolius*, but its flowers are more reminiscent of *L. sylvestris*.

**Figure 5.**
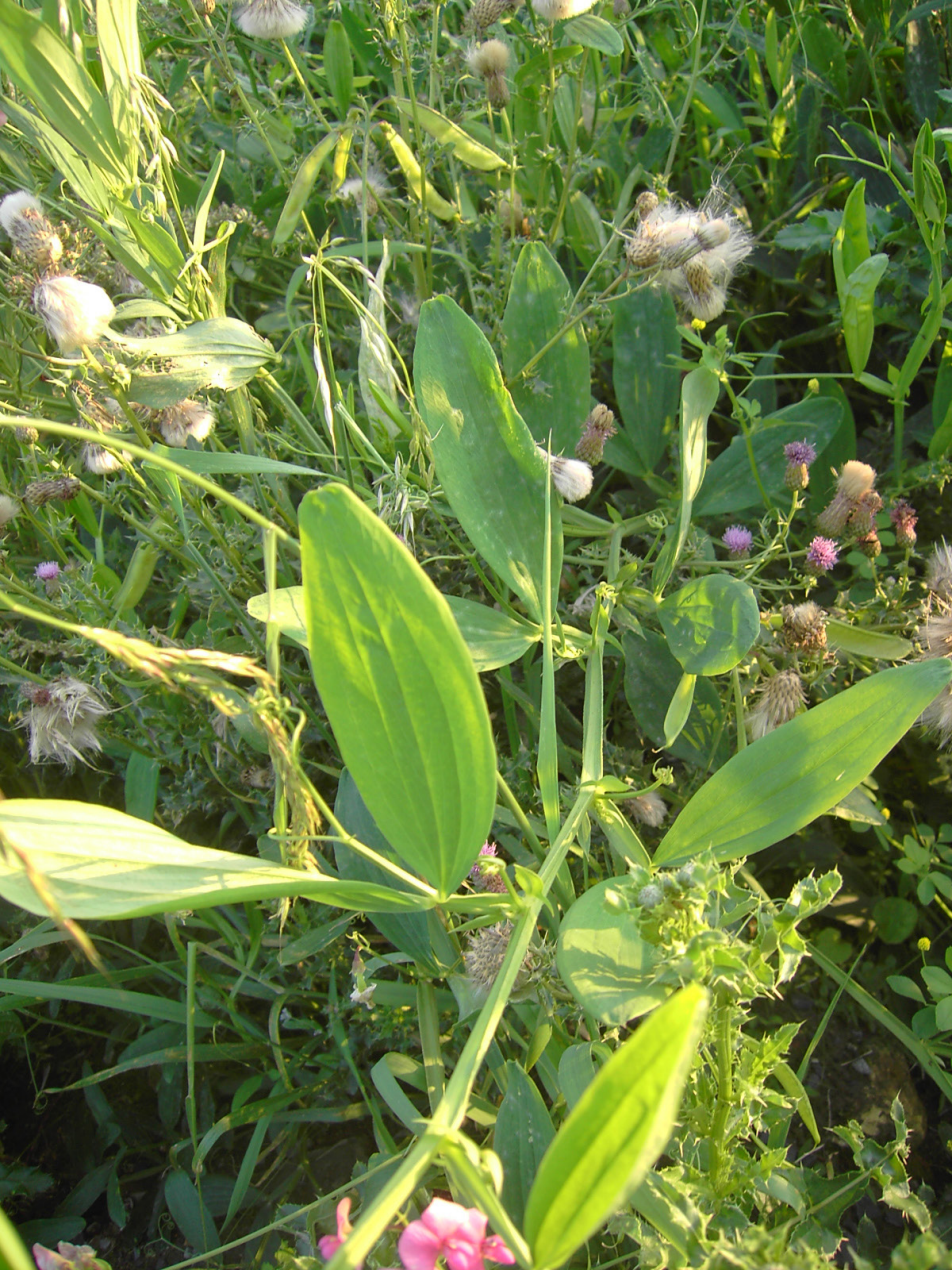
*
Lathyrus platyphyllos* in Kortemark, Belgium, 1 September 2011, F. Verloove. Leaflets of mid-stem leaves are broad and rounded-mucronate at the apex.

**Figure 6.**
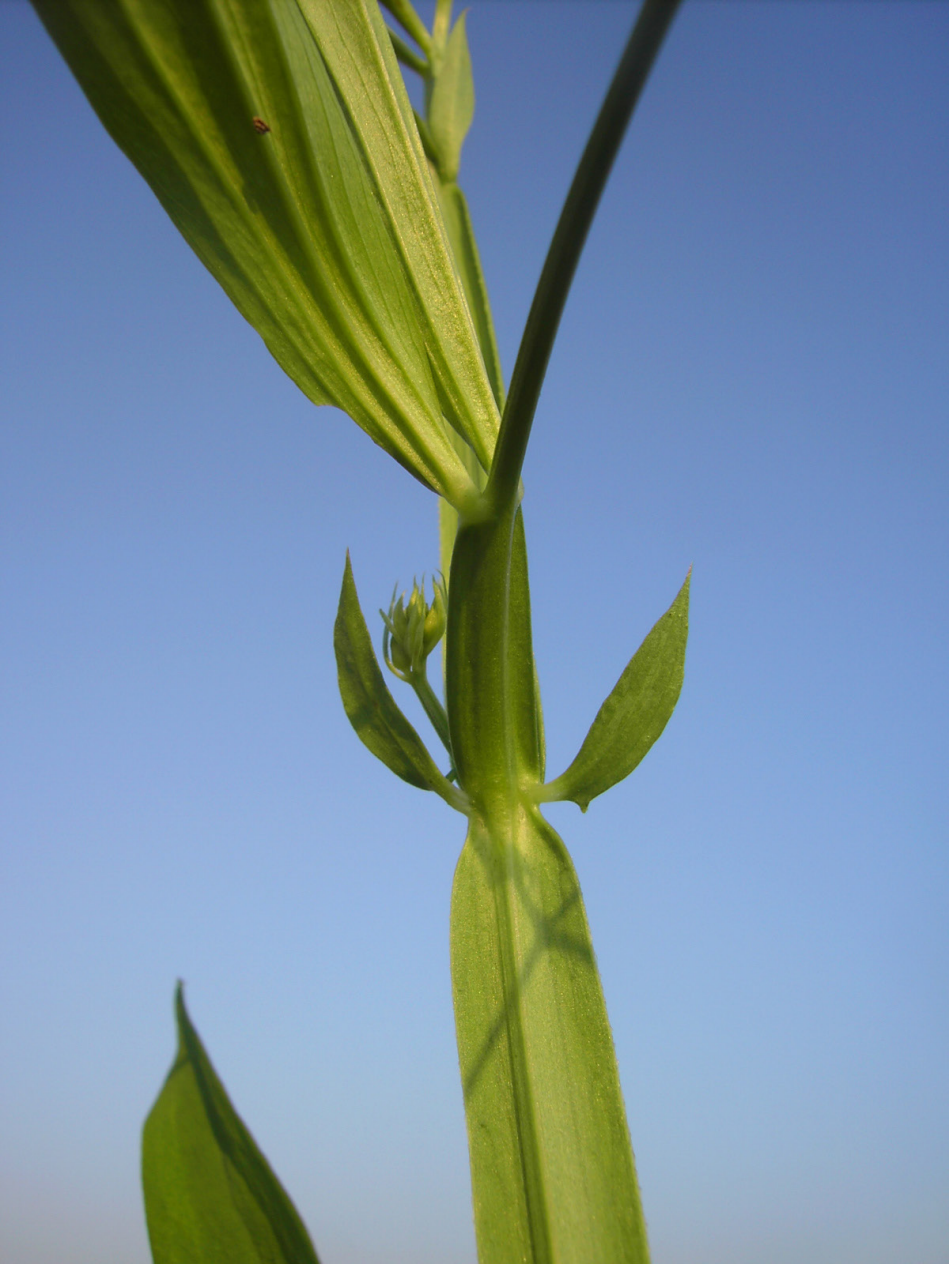
*
Lathyrus platyphyllos* in Kortemark, Belgium, 1 September 2011, F. Verloove. Stipules are wider than in *L. sylvestris*, yet narrower than the stem. Stem wings also tend to be wider than in *L. sylvestris*, resembling those of *L. latifolius*.

**Figure 7.**
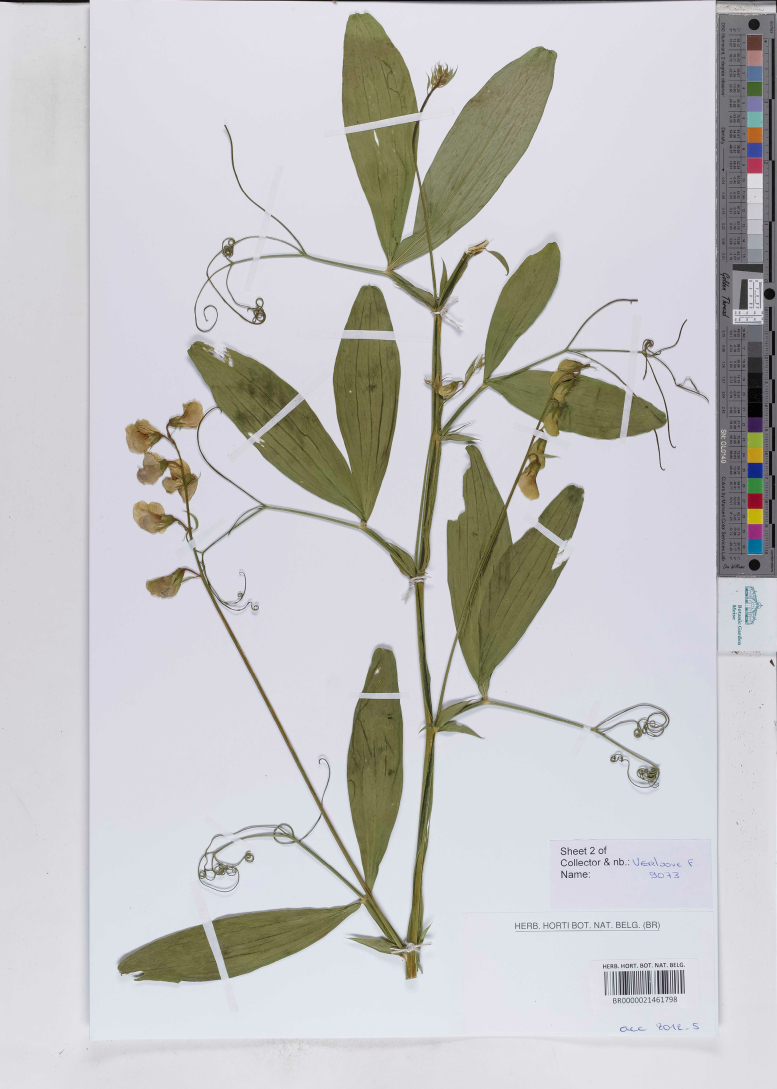
Herbarium specimen of *Lathyrus platyphyllos* collected in 2011 in Kortemark, Belgium and preserved in BR (BR0000021461798). This specimen was included in the genetic analyses.

Species resembling *Lathyrus sylvestris* in general habit, but differing in several vegetative characters:

Leaflets of mid-stem leaves are considerably broader (up to 40 mm wide), with a length-to-width ratio of ca. 3.5–4(–5):1 (vs. ca. 8–9:1 in *L. sylvestris*). Leaflet apices are usually broadly rounded to truncate or sometimes slightly emarginate and mucronate (Figs 4, 5, 7), rather than sharply acute and long-tapering (Figs 13, 14). Stipules tend to be slightly broader, reaching up to 4 mm in width (vs. typically ≤ 2 mm in *L. sylvestris*) and the stem wings are likewise wider, up to 3 mm (vs. rarely exceeding 1 mm in *L. sylvestris*) (Fig. 6).

##### Revised herbarium material.

(see Verloove et al. (2025), Suppl. material 1: table S1).

##### Notes.

Within the *Lathyrus sylvestris-latifolius* complex, numerous diagnostic characters have been proposed over time, the taxonomic value of which has often proven limited. Variation in stem and petiole wing width, for instance, may occur even within a single individual. Similarly, the relative length and equality of the calyx teeth— whether approximately equal or distinctly unequal— appears highly variable and unreliable for diagnostic purposes. Calyx teeth tend to be shortest and least unequal in *L. sylvestris*, but exhibit a gradual transition towards the other species.

In its typical form, *L. heterophyllus* is readily separated from the remaining taxa by the presence of two (rarely three) pairs of leaflets on most leaves, particularly those of the mid- and upper stem, whereas the other species consistently bear only a single pair.

Unijugate forms of *L. heterophyllus* (‘var. *unijugus*’) have long complicated the delimitation of *L. platyphyllos*. Both are exceedingly similar in vegetative and floral morphology, as already noted by Koch (1843). According to Sell and Murrell (2009), the only reliable distinguishing character lies in the relative width of the stipules compared to the stem— an admittedly labile trait within this complex. Gams (1924), in *Illustrierte Flora von Mitteleuropa*, likewise cited this feature, together with several others of even lesser diagnostic value. Examination of Swedish specimens with unijugate leaves confirms that the stipules are conspicuously broad, consistently exceeding the stem in width and that the hilum of the seed encircles at most one-third of the seed circumference. This latter feature, in particular, appears stable and may, therefore, retain some discriminative significance.

By contrast, *L. latifolius* differs clearly from both *L. sylvestris* and *L. platyphyllos* in its larger, more vividly pink flowers, the style being hairy over a greater portion of its length and its broad stipules (up to 15 mm wide, consistently broader than the stem). Its calyx teeth are strongly unequal, the longest clearly exceeding the calyx tube; the hilum of the seed likewise encircles at most one-third of the circumference.

Taken together, *L. sylvestris* and *L. platyphyllos* are readily separable from *L. heterophyllus* and *L. latifolius*, yet they remain much more difficult to distinguish from one another. Only a few characters appear consistently reliable: chiefly, the relative width and shape of the mid-stem leaflets and their apices, as well as the tendency for slightly broader stipules and stem wings in *L. platyphyllos*.

##### Distribution.

As *Lathyrus platyphyllos* is a scarcely known species, information on its natural distribution remains limited. It was described from Denmark and historical sources additionally indicate that it is native to Central Europe (Germany, Austria, Czechia and Switzerland) and possibly also to eastern France (Jura), Poland (Silesia) (Gams 1924) and northern Italy (Fiori 1925). According to Gams (1924), the species is occasionally cultivated as an ornamental. The natural distribution of *L. platyphyllos* is entirely embedded within the broader range of *L. sylvestris*, which is much more widespread across Europe.

In Belgium, by contrast, *L. platyphyllos* is almost certainly not native. It was first reliably recorded only in 1943 and is confined to habitats subject to varying degrees of human disturbance, most commonly along railway lines or on disused railway grounds. The species is naturalised in a few widely scattered localities, particularly in the broader regions of Antwerp and Ghent—where it may be locally frequent—and in central West Flanders, with additional, more isolated populations elsewhere in the country.

### Identification key

The following key distinguishes the four species from the *Lathyrus sylvestris-latifolius* complex as treated in this study.

**Table:** 

1	At least some leaves with two or three pairs of leaflets (Fig. 8)	*** Lathyrus heterophyllus***
–	All leaves with a single pair of leaflets	**2**
2	Corolla 20–30 mm long, magenta (paler in cultivars), standard not or rarely suffused with green (Fig. 9); style pubescent for 3–5 mm from apex; stipules up to 15 mm wide, always much broader than the stem (excl. wings) (Figs 10, 11)	*** L. latifolius***
–	Corolla 15–20 mm long, pinkish-purple, standard often suffused with green (Fig. 9); style pubescent for 1.5–2.8 mm from apex; stipules usually narrower, at most as wide as the stem (excl. wings) or clearly wider than it	3
3	Stipules conspicuously wider than the stem (Fig. 12); hilum of seed encircling at most one-third of its circumference	*** L. heterophyllus***
–	Stipules at most as wide as the stem; hilum of seed encircling about half of the circumference	**4**
4	Leaflets narrow, ca. 8–9× as long as wide, tapering to a long, acute apex (Figs 13, 14); stipules 1–2 mm wide; stem wings ca. 1 mm wide	*** L. sylvestris***
–	Leaflets of mid-stem leaves broader, ca. 3.5–4(–5)× as long as wide, apices usually broadly rounded or emarginate, mucronate (Figs 4, 5, 7); stipules up to 4 mm wide; stem wings up to 3 mm wide (Fig. 6)	*** L. platyphyllos***

**Figure 8.**
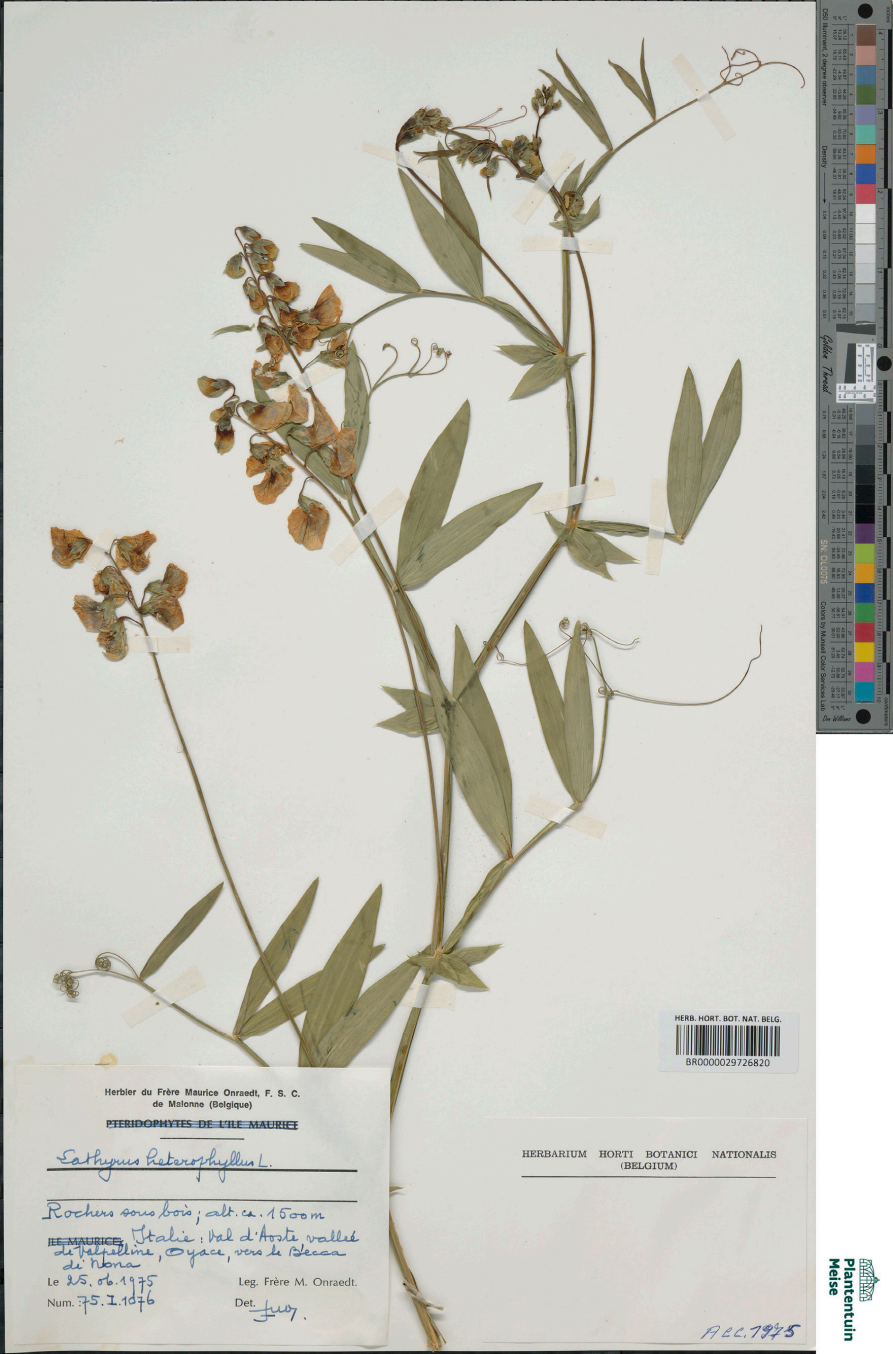
Herbarium specimen of *Lathyrus heterophyllus* collected in 1975 in Italy and preserved in the BR herbarium (BR0000029726820). In its typical form, leaves bear two pairs of leaflets. Stipules are broad and exceed the stem in width. This specimen was included in the genetic analyses.

**Figure 9.**
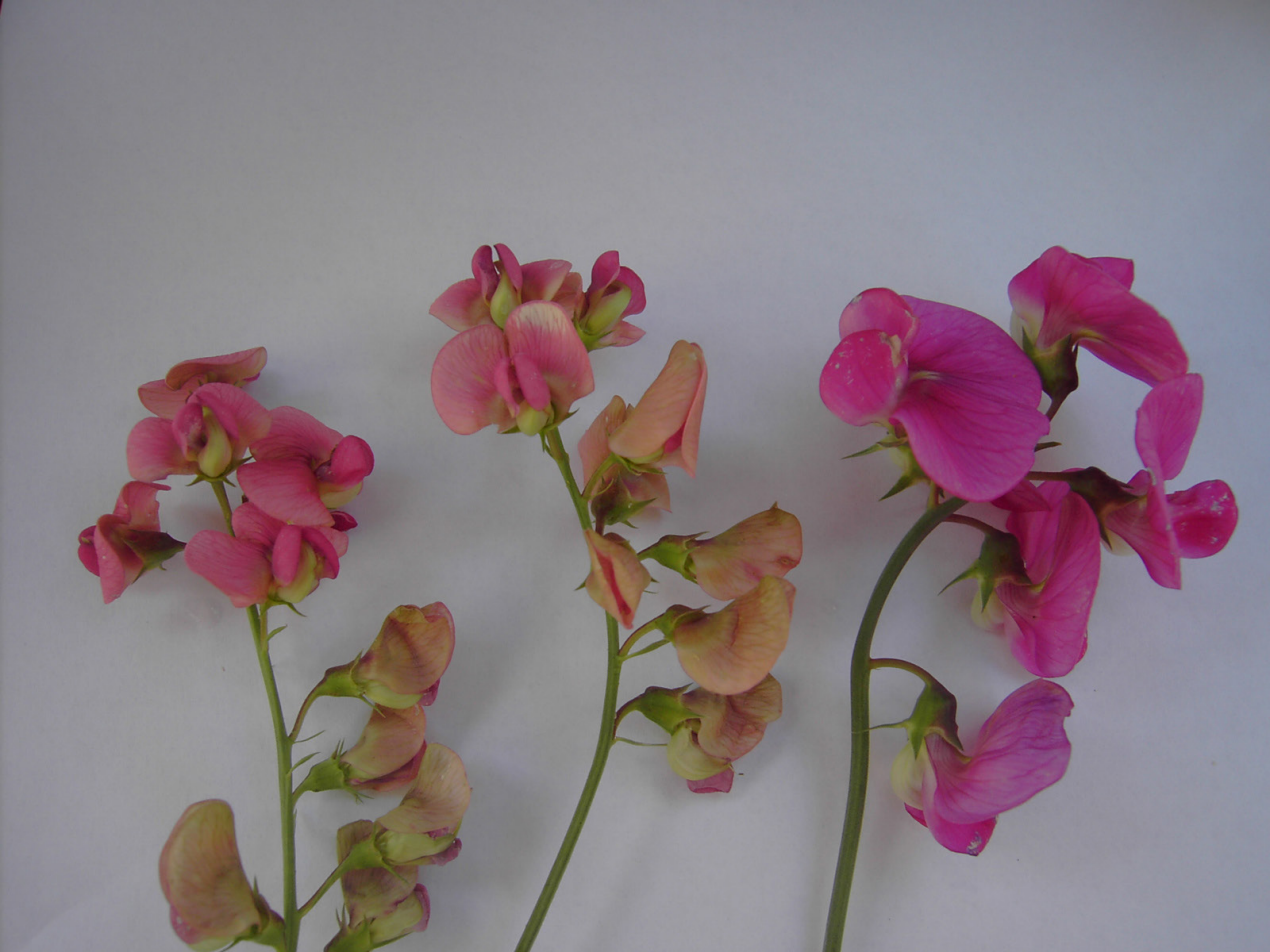
Flowers of *Lathyrus platyphyllos* (two branches on the left) and *L. latifolius* (one branch on the right) for comparison, 3 September 2011, F. Verloove. Flowers of *L. platyphyllos* are markedly smaller and less vivid in colour.

**Figure 10.**
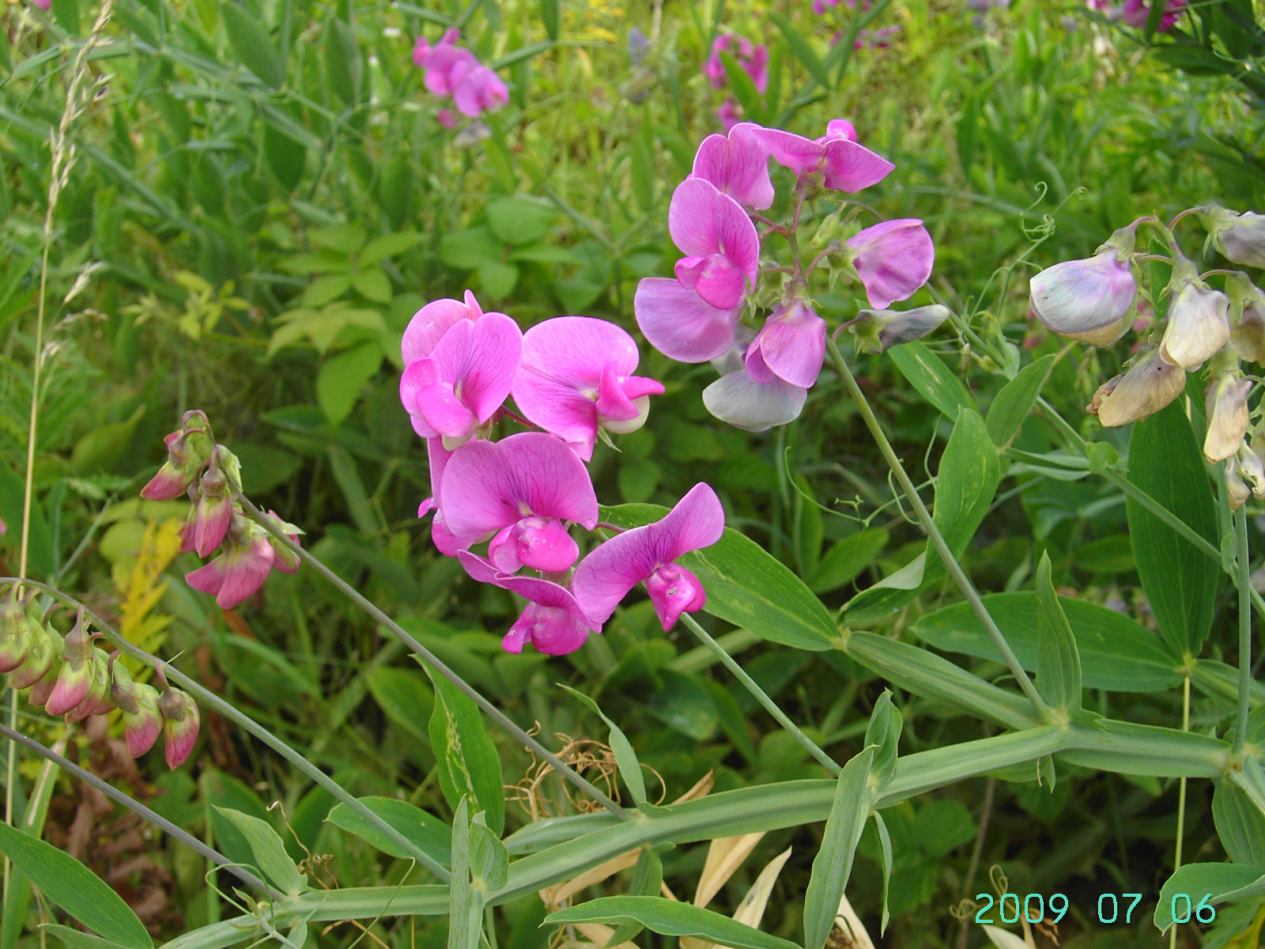
*
Lathyrus latifolius* in Ieper, Belgium, 6 July 2009, F. Verloove. Compared with *L. platyphyllos*, the flowers are larger and more vividly coloured. Amongst the species examined, *L. latifolius* has the broadest stipules.

**Figure 11.**
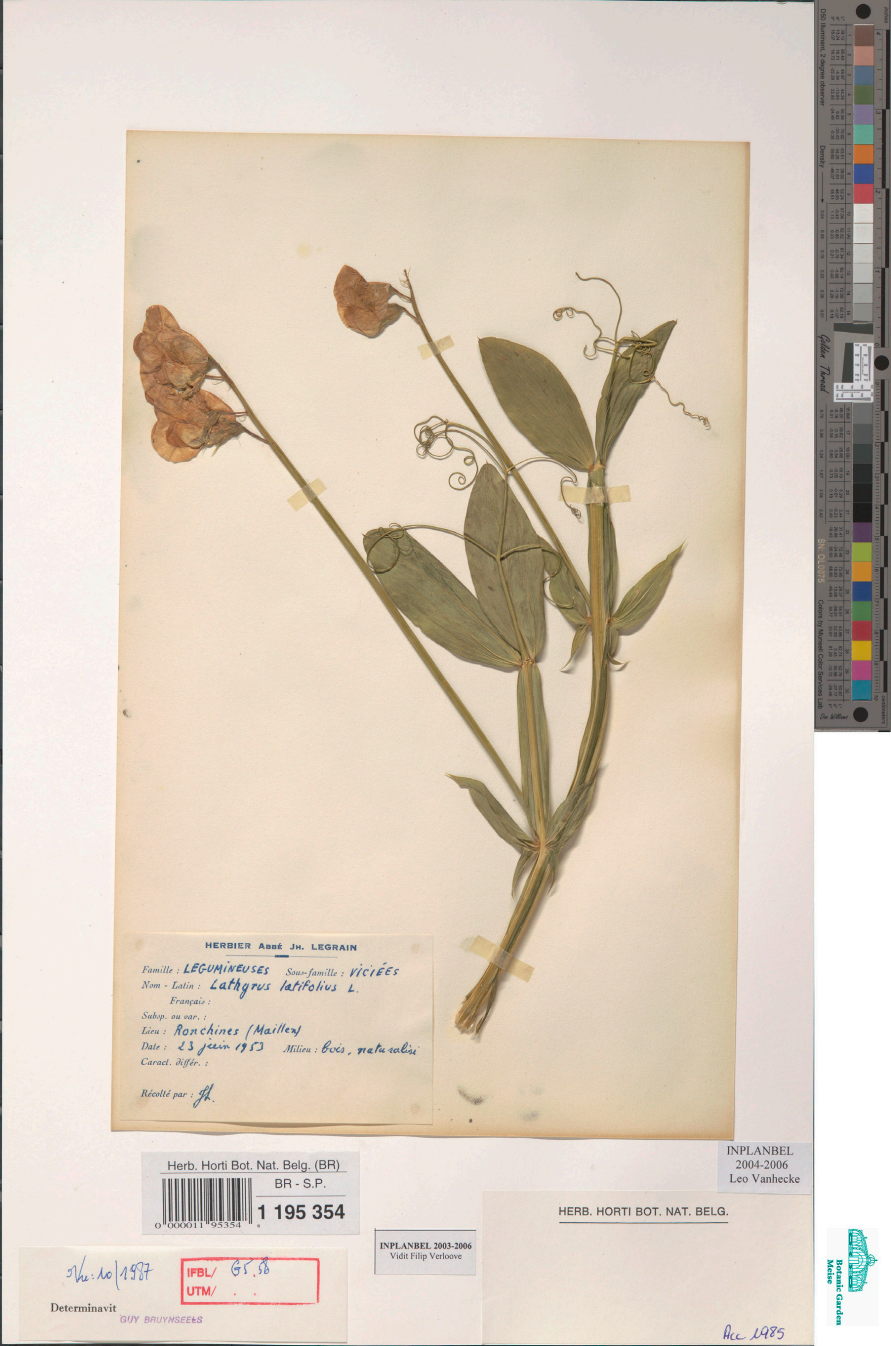
Herbarium specimen of *Lathyrus latifolius* collected in 1953 in Ronchines, Belgium and preserved in BR (BR0000011953548). This specimen was included in the genetic analyses.

**Figure 12.**
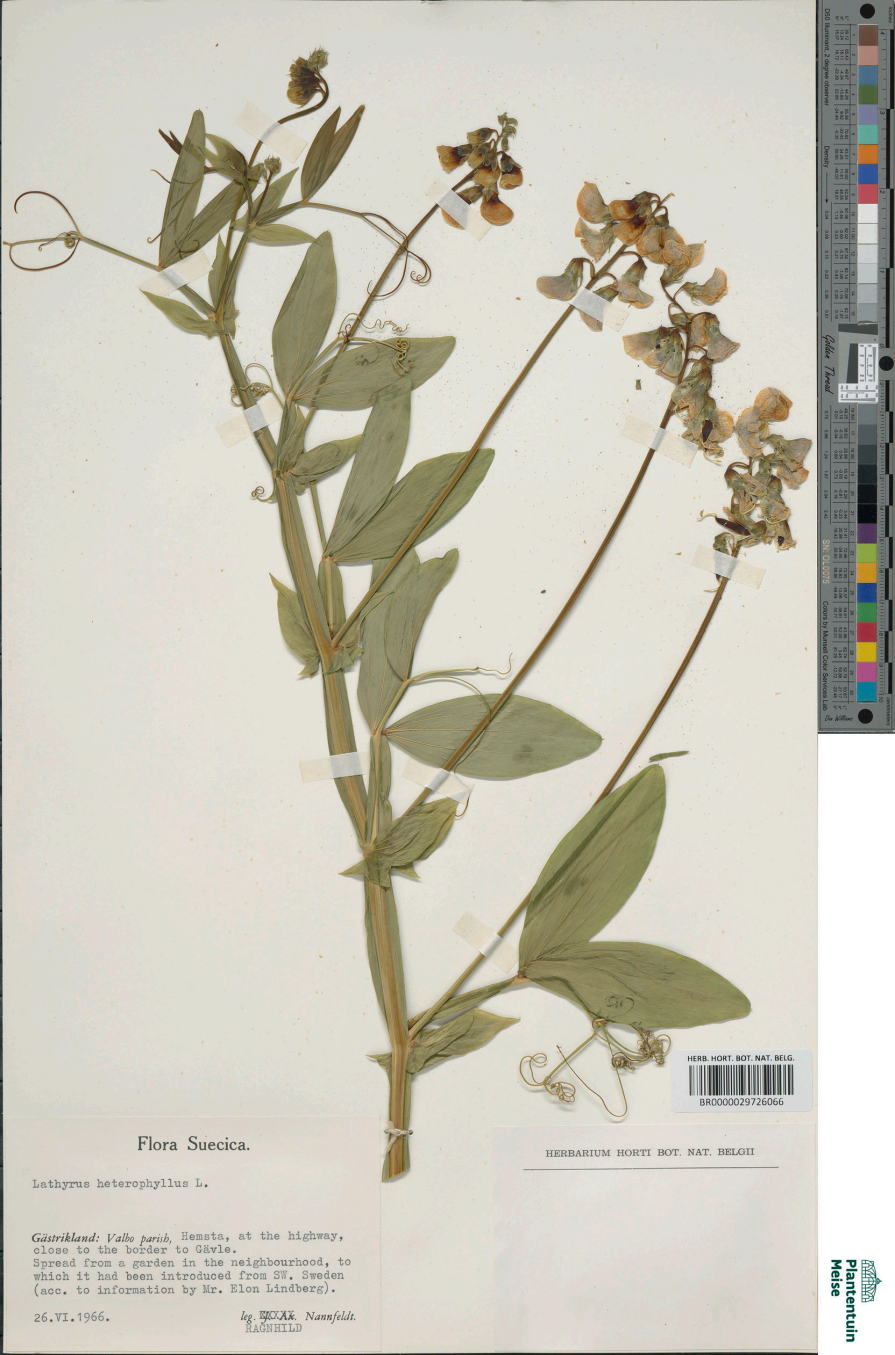
Herbarium specimen of *Lathyrus heterophyllus* collected in Sweden in 1966 and preserved in BR (BR0000029726066). Scandinavian plants usually bear only one pair of leaflets (“var. *unijugus*”), but were not differentiated genetically from plants with two pairs. Such unijugate plants resemble *L. platyphyllos*, but can be separated, amongst other characters, by their much broader stipules. This specimen was included in the genetic analyses.

**Figure 13.**
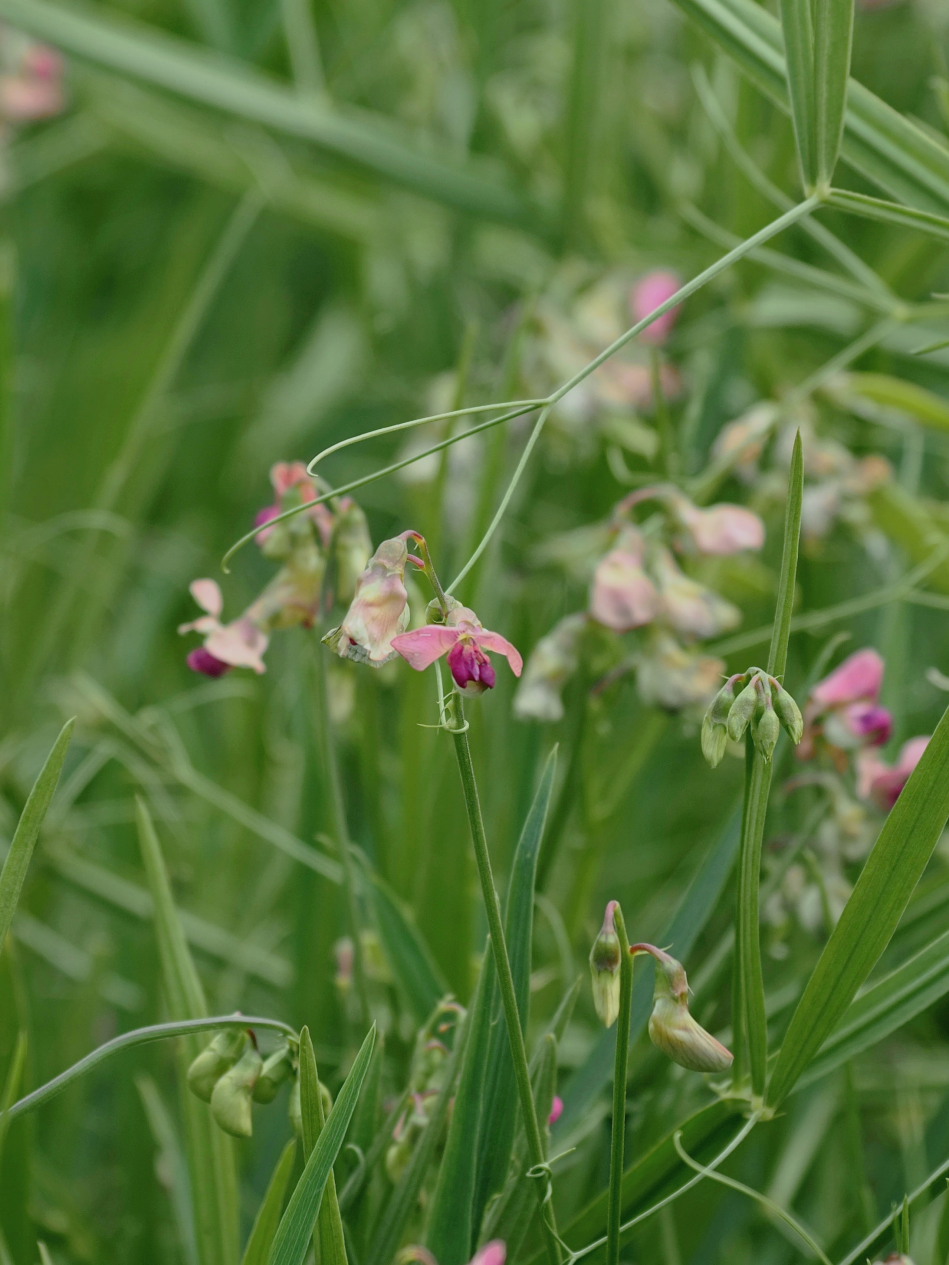
*
Lathyrus sylvestris* in Ostend, Belgium, 26 August 2025, L. Devos. Leaflets are very narrow, with a sharply-pointed, long-acuminate apex. Flowers are comparable to those of *L. platyphyllos* in both size and colour.

**Figure 14.**
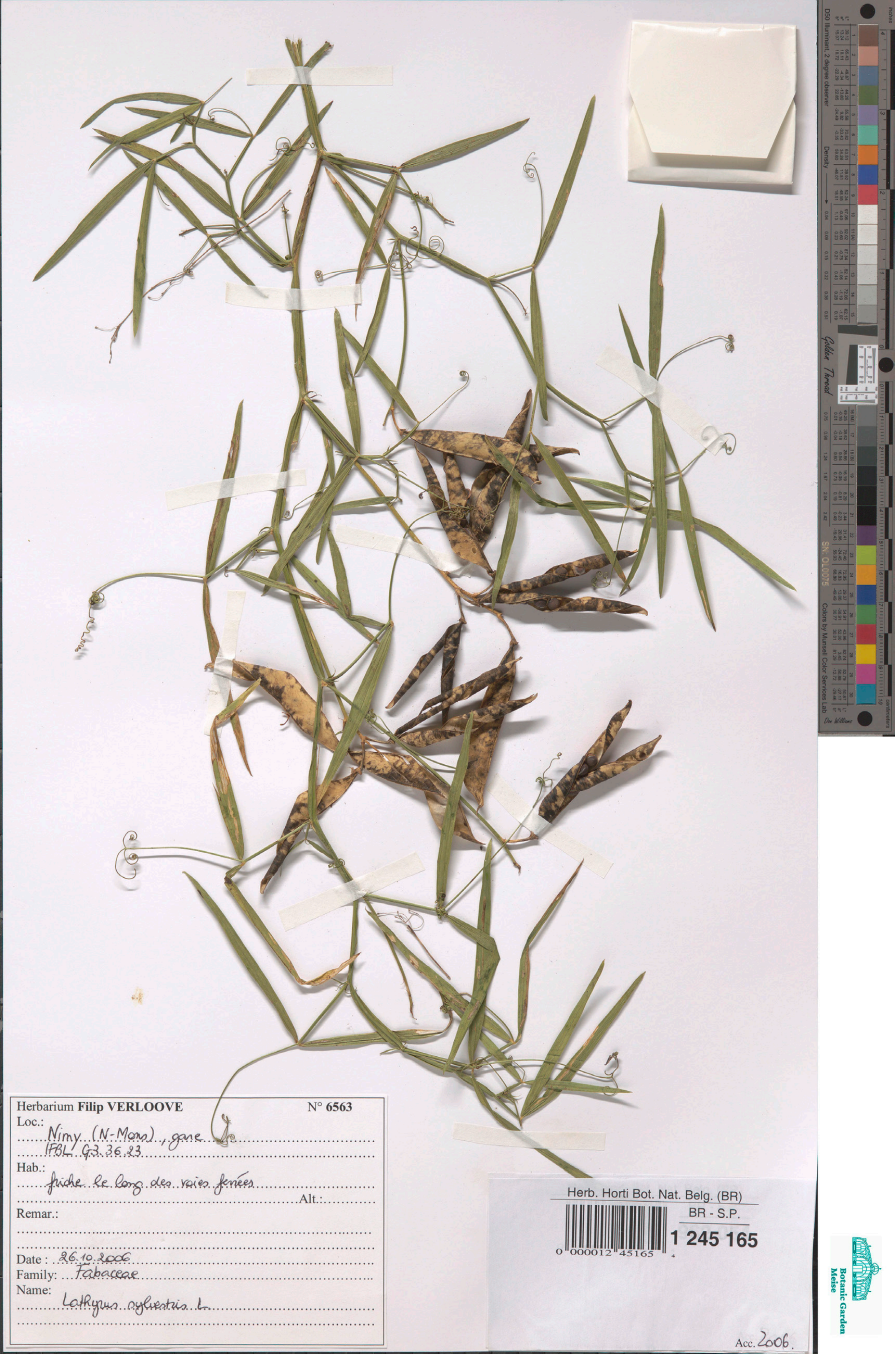
Herbarium specimen of *Lathyrus sylvestris* collected in 2006 in Nimy, Belgium and preserved in BR (BR0000012451654). This specimen was included in the genetic analyses.

### Conclusions

Our results demonstrate that the *Lathyrus* indet. specimens, previously treated under *Lathyrus sylvestris* var. platyphyllos or *L. heterophyllus* var. *unijugus*, represent a genetically distinct lineage. Phylogenetic and distance-based analyses show that this taxon is more closely related to *L. sylvestris* than to *L. heterophyllus*, yet genetically divergent from both. Contrary to current taxonomic consensus, these findings suggest that this variant does hold taxonomic significance. In contrast, the unijugate form, traditionally known as *L. heterophyllus* var. *unijugus*, is genetically indistinguishable from typical *L. heterophyllus*, indicating that its morphological variation reflects intraspecific plasticity rather than true taxonomic differentiation. The Belgian taxon is, therefore, unrelated to var. *unijugus* and warrants recognition under the reinstated name *Lathyrus platyphyllos* (Retz.) W.D.J.Koch. This reinstatement provides a coherent framework for interpreting morphological and genetic variation within the *L. sylvestris*-latifolius complex and facilitates accurate monitoring and management of non-native populations, which appear to be naturalising and potentially invasive in Belgium.

## Supplementary Material

XML Treatment for Lathyrus platyphyllos

## Additional information

### Use of AI

No use of AI was reported.

### Author ORCIDs

Filip Verloove https://orcid.org/0000-0003-4144-2422

Sander de Backer https://orcid.org/0000-0001-8891-1519

Yves Bawin https://orcid.org/0000-0002-1663-6535

Quentin Groom https://orcid.org/0000-0002-0596-5376

Frederik Leliaert https://orcid.org/0000-0002-4627-7318

Sofie Meeus https://orcid.org/0000-0003-0715-8647

Steven B. Janssens https://orcid.org/0000-0003-1246-9474

### Data availability

The supplementary data that support the findings of this study are compiled and openly available in Zenodo at https://doi.org/10.5281/zenodo.18962465. Specimens used in this study have been digitised and can be found on GBIF at https://doi.org/10.15468/DL.USZHJM and BotanicalCollections.be. The scripts used in this paper are available via Zenodo at https://doi.org/10.5281/zenodo.17648190 (variant filtering) and GitLab at https://gitlab.com/ybawin/smapapps (SMAPapps).

## Supplementary materials

Supplementary material 1 Supplementary tables and figures https://doi.org/10.3897/phytokeys.273.180869.suppl1 Data type zip Explanation note **table S1**. Revised herbarium material.csv: Herbarium specimens (N=71) metadata included in this study. This file is based on the GBIF download (https://doi.org/10.15468/DL.USZHJM). **table S2**. SpeciesDelimitationAnalyser result.csv: Species delimitation results from SpeciesDelimitationAnalyser. **fig. S1**. single_locus_trees: Maximum-likelihood (IQ-Tree) trees inferred from the ITS, trnS-G, trnL-F, rbcL, and trnH-psbA markers, showing the Lathyrus indet. clade. UF bootstrap values (≥90%) are indicated at each branch. Meise Botanic Garden herbarium barcodes (BR) are indicated before the taxon names. **fig. S2**. Jaccard_Inversed_genetic_distance_heatmap_annot.svg: Matrix of inverse Jaccard distances calculated from haplotype calls. Haplotype frequencies were first discretized into dominant presence/absence calls (0 or 1) using a frequency bound interval of 20%. Based on these discrete haplotype calls, pairwise Jaccard similarity coefficients (J) were computed following Jaccard (1912). The resulting matrix reflects haplotype composition similarity across samples. **fig. S3**. SNAPP.png: Species tree inferred using SNAPP v1.6.1 in BEAST v2.7.7. Branch support is indicated by posterior probability values. **references.bib**: A BiBTex file containing all the references used in the study. https://binary.pensoft.net/file/1603963This dataset is made available under the Open Database License (http://opendatacommons.org/licenses/odbl/1.0/). The Open Database License (ODbL) is a license agreement intended to allow users to freely share, modify, and use this Dataset while maintaining this same freedom for others, provided that the original source and author(s) are credited. Filip Verloove, Sander de Backer, Yves Bawin, Quentin Groom, Frederik Leliaert, Sofie Meeus, Steven B. Janssens
