# Supplementary material for: Morphological and genomic data from Belgian naturalised populations reinstate the enigmatic Lathyrus platyphyllos (Fabaceae) as a distinct European species
Source: PhytoKeys. 2026 Apr 23;273:255–79. doi: 10.3897/phytokeys.273.180869 (PMC13133675; doi:10.3897/phytokeys.273.180869)
Supplement: Supplementary material 1 — Supplementary tables and figures [file phytokeys-273-255_article-180869__-s001.zip › Fig_S1_single_locus_trees.pdf]

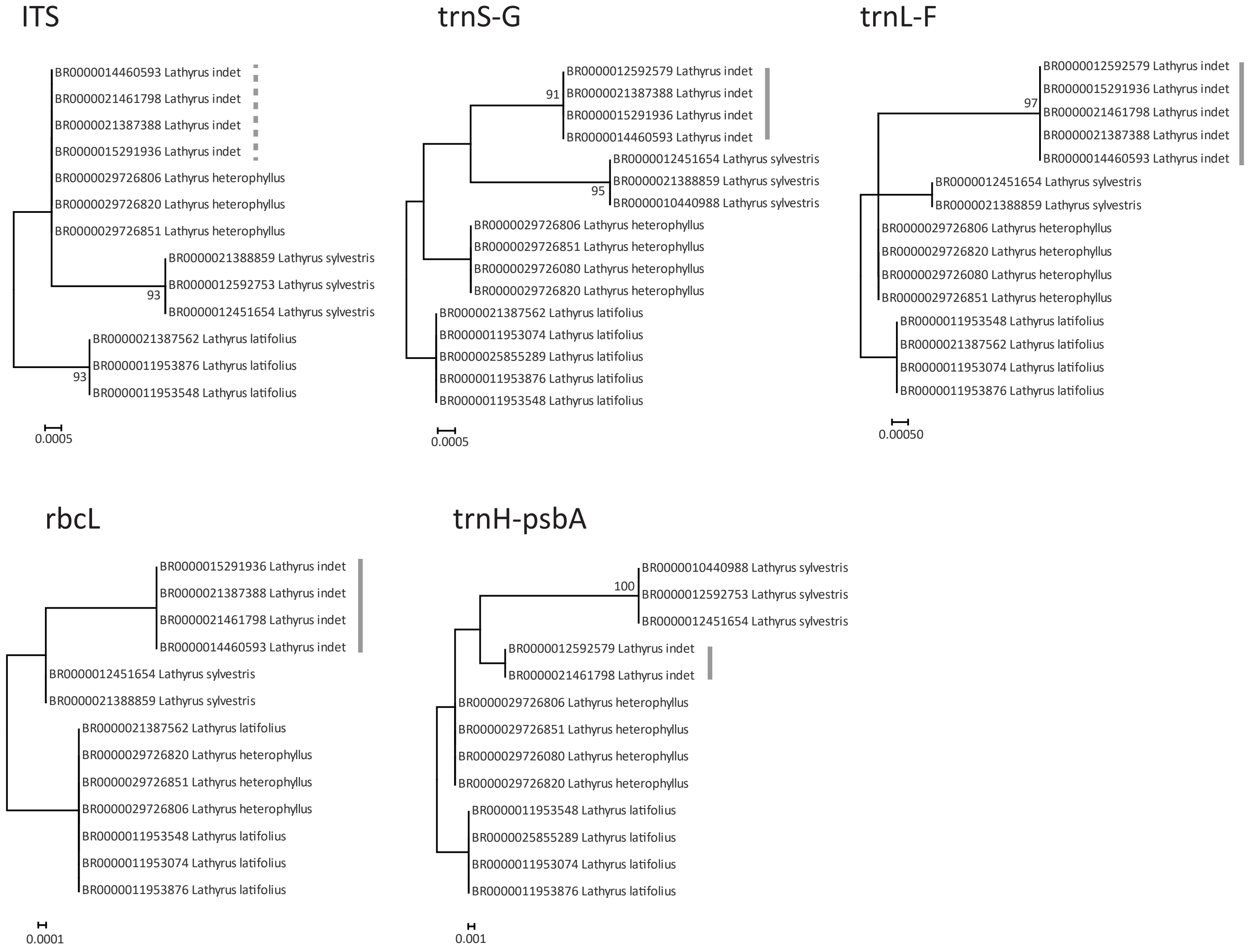

Fig. S1. Maximum-likelihood (IQ-Tree) trees inferred from the ITS, trnS-G, trnL-F, rbcL, and trnH-psbA markers, showing the *Lathyrus* indet. clade. UF bootstrap values (≥90%) are indicated at each branch. Meise Botanic Garden herbarium barcodes (BR) are indicated before the taxon names.
