# Supplementary figures and images for: Morphological and genomic data from Belgian naturalised populations reinstate the enigmatic Lathyrus platyphyllos (Fabaceae) as a distinct European species
Source: PhytoKeys. 2026 Apr 23;273:255–79. doi: 10.3897/phytokeys.273.180869 (PMC13133675; doi:10.3897/phytokeys.273.180869)

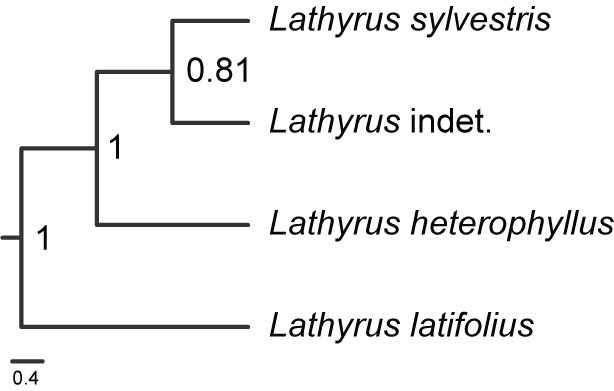

Supplement: Supplementary material 1 — Supplementary tables and figures [file phytokeys-273-255_article-180869__-s001.zip › Fig_S3_SNAPP (1).png]
